# Bisphenol S Impairs Oestradiol Secretion during In Vitro Basal Folliculogenesis in a Mono-Ovulatory Species Model

**DOI:** 10.3390/toxics10080437

**Published:** 2022-07-30

**Authors:** Claire Vignault, Véronique Cadoret, Peggy Jarrier-Gaillard, Pascal Papillier, Ophélie Téteau, Alice Desmarchais, Svetlana Uzbekova, Aurélien Binet, Fabrice Guérif, Sebastien Elis, Virginie Maillard

**Affiliations:** 1 CNRS, IFCE, INRAE, Université de Tours, PRC, 37380 Nouzilly, France; claire.vignault@inrae.fr (C.V.); veronique.cadoret@inrae.fr (V.C.); peggy.jarrier-gaillard@inrae.fr (P.J.-G.); pascal.papillier@inrae.fr (P.P.); teteau.ophelie@orange.fr (O.T.); alice.desmarchais@inrae.fr (A.D.); svetlana.uzbekova@inrae.fr (S.U.); aurelien.binet@inrae.fr (A.B.); fabrice.guerif@univ-tours.fr (F.G.); sebastien.elis@inrae.fr (S.E.); 2 Service de Médecine et Biologie de la Reproduction, CHRU de Tours, 37000 Tours, France; 3 Service de Chirurgie Pédiatrique Viscérale, Urologique, Plastique et Brûlés, CHRU de Tours, 37000 Tours, France

**Keywords:** ovary, endocrine disruptors, follicular growth, hormonal secretions, gene expression, bisphenols, plasticiser, ewe

## Abstract

Bisphenol S (BPS) affects terminal folliculogenesis by impairing steroidogenesis in granulosa cells from different species. Nevertheless, limited data are available on its effects during basal folliculogenesis. In this study, we evaluate in vitro the effects of a long-term BPS exposure on a model of basal follicular development in a mono-ovulatory species. We cultured ovine preantral follicles (180–240 μm, *n* = 168) with BPS (0.1 μM (possible human exposure dose) or 10 μM (high dose)) and monitored antrum appearance and follicular survival and growth for 15 days. We measured hormonal secretions (oestradiol (at day 13 [D13]), progesterone and anti-Müllerian hormone [D15]) and expression of key follicular development and redox status genes (D15) in medium and whole follicles, respectively. BPS (0.1 µM) decreased oestradiol secretion compared with the control (−48.8%, *p* < 0.001), without significantly impairing antrum appearance, follicular survival and growth, anti-Müllerian hormone and progesterone secretion and target gene expression. Thus, BPS could also impair oestradiol secretion during basal folliculogenesis as it is the case during terminal folliculogenesis. It questions the use of BPS as a safe BPA substitute in the human environment. More studies are required to elucidate mechanisms of action of BPS and its effects throughout basal follicular development.

## 1. Introduction

Folliculogenesis is a long and discontinuous developmental process that leads to the ovarian follicle growth and that requires constant tight communications between oocyte and somatic follicle cells (granulosa, cumulus and theca cells) [1,2]. From a functional perspective, follicular development is divided into two successive phases: basal folliculogenesis corresponds to the initial development of primordial follicles released from the ovarian reserve to an antral follicle. It is a gonadotrophin hormone independent phase during which granulosa cells have an intense mitotic activity and the ability to produce Anti-Müllerian Hormone (AMH) and some steroid hormones. In contrast, terminal folliculogenesis, which leads to ovulation of a mature and competent oocyte, is strictly dependent on Follicle-Stimulating Hormone (FSH) and Luteinising Hormone (LH) presence. During this phase, granulosa cell proliferation and AMH production decrease, whereas steroidogenesis activity drastically increases. Folliculogenesis is regulated by endogenous growth factors, cytokines, gonadotropins and steroid hormones as well as exogenous factors, such as nutrients and environmental factors [3]. Thus, several environmental pollutants (e.g., the pesticide dichlorodiphenyltrichloroethane (DDT), the fungicide vinclozolin, the synthetic oestrogen Diethylstilbestrol (DES), the plasticiser bisphenol A (BPA), etc.) that present endocrine-disrupting properties can indeed affect female fertility by altering ovarian development and functions [4].

Over the last 20 years, BPA has become one of the most studied endocrine disruptors due to its massive worldwide use in many everyday plastic materials, mainly in polycarbonate plastics (for food containers, cosmetics, electronics, etc.) and epoxy resins (as a protective coating for food cans, pipes, floors and as composite in paints, etc.) [5]. Therefore, BPA human exposure occurs mainly through contaminated diet [6,7] but also through inhalation of plastic dust [8,9] and percutaneous absorption [10,11]. Based on its endocrine-disrupting properties and its possible involvements in the development of human pathologies (metabolic and reproductive disorders and cardiovascular diseases, among others) [12,13,14,15,16], BPA use has been regulated in some countries from the European Union and in Canada [17,18]. Consequently, BPA has been replaced with several structural analogues, with bisphenol S (BPS) being the most used [19,20].

Similar to BPA, BPS is nowadays detected in the environment [9,19] and in several human body fluids and tissue, including urine [21,22,23], plasma or serum, [24,25], hair [26] and in follicular fluid [27]. Thus, BPS can be in contact with ovarian follicular cells and can induce female mammalian reproductive dysfunctions [28,29]. Indeed, BPS altered oocyte quality and/or embryo development in sows [30], cows [31,32], mice [33,34,35] and ewes [36]. Furthermore, BPS impaired ovarian steroidogenesis in vivo or in vitro from granulosa cells of both mono-ovulatory species (bovine [31], ovine [37] and human [27]) and poly-ovulatory species (swine [38,39] and rodents [40,41]). Most of these studies have focussed on terminal folliculogenesis and have highlighted differences in BPS effects depending on species, BPS doses and exposure time. There are few studies on the effects of BPS on basal folliculogenesis. These studies have been conducted in rodent models after in vivo BPS exposure; one of them has shown an increased number of primordial follicles [35] and others have reported a decreased number of preantral and/or antral follicles [33,35,42,43]. These data suggest a potential action of BPS on primordial follicle and antrum formation in rodents, but no data are available in other species or in mono-ovulatory species in particular.

The mechanisms of action of bisphenols are not yet fully understood in the gonads. Studies have shown that bisphenols could interact with several receptor-mediated signalling pathways in different cell types—for example, nuclear Estrogen Receptors (ERα and ERβ), Estrogen-Related Receptors (ERRγ) and Aryl hydrocarbon Receptor (AhR) [44,45,46,47]. They could also act by altering DNA methylation in different cells [48,49,50,51]. Finally, bisphenols could also enhance oxidative stress in different cells [52,53,54], including granulosa cells [38,55]. However, to date there is scarce information regarding the potential mechanisms of action of BPS during basal folliculogenesis.

We hypothesized that BPS could impair basal follicular growth and antrum appearance in a mono-ovulatory species. We aimed then to study BPS effects on follicular development and hormonal secretions during basal folliculogenesis (especially in the antrum-appearance phase), as it has already been described for terminal folliculogenesis. We chose the ewe as a study model because its ovarian development and folliculogenesis duration present similarities with those of the human species [2,56,57]. Thus, we studied the effects of two concentrations of BPS on ewe basal follicular development in vitro, especially on follicular growth and survival, antral cavity appearance, follicle hormonal secretions (oestradiol, progesterone and Anti-Müllerian Hormone (AMH)) and the expression of key genes in follicular development and redox status.

## 2. Materials and Methods

Unless stated otherwise, all culture media and chemicals used in the present study were purchased from Merck Sigma-Aldrich (Saint-Quentin-Fallavier, France).

### 2.1. Collection of Ovaries and Isolation and In Vitro Culture of Preantral Follicles

Over 300 ovaries were recovered from peri-pubertal ewes (over 150 animals) at a local commercial slaughterhouse to collect all 168 healthy follicles used in the seven independent experiments of this study. Ovaries were washed with sterile 0.9% NaCl supplemented with 52.35 µM gentamicin, and then transported to the laboratory within 2 h after collection in tubes containing HEPES-buffered tissue culture medium 199 supplemented with 6 µM bovine serum albumin (BSA) and 52.35 µM gentamicin (TCM199+). The ovaries were cut into thin slices using a sterile surgical blade. These slices were incubated at 37 °C for 1 h in a cell dissociation phosphate-buffered saline (PBS)-based solution containing 0.1% collagenase IA (*m*/*v*) and 0.01% DNase I (*m*/*v*), and then in a stop solution of PBS containing 0.3 mM BSA. After rinsing in warm TCM199+ medium, follicles were mechanically isolated from the cortical ovary slices by micro-dissection under a stereomicroscope using two 30-gauge needles fitted to 1 mL syringe barrels. Subsequently, the follicles were stored in Petri dishes containing TCM199+ medium. On this day 0 (D0), the initial diameter of all follicles was measured on the perpendicular axes with a stereomicroscope equipped with a calibrated ocular micrometer. Only healthy preantral follicles between 180 μm and 240 μm in diameter, with no apparent damage to the basal membrane, no visible signs of degeneration (darkness of the oocyte and follicular cells) and no antral cavity were selected for culture.

The follicle culture was adapted from a previously described protocol [58]. The culture medium (MEM+) was prepared the day prior to the culture (or medium renewal) with sodium-bicarbonate-buffered Minimum Essential Medium Eagle (MEM; alpha modification) supplemented with 2 mM glutamine, 2 mM hypoxanthine, 0.28 mM ascorbic acid and ITS+ Universal Culture Supplement Pre-mix (1.08 μM insulin, 81.1 nM transferrin, 48.5 nM selenium, 18.8 µM BSA and 19.1 µM linoleic acid; Corning, D. Dutscher, Issy-les-Moulineaux, France) and with different concentrations of BPS (0, 0.1 or 10 μM). The 0.1 µM dose was chosen, because such concentrations were reported in human biological fluids (urine and plasma) for some people in several studies [19,25,59]. Thus, we decided to name this concentration a possible human exposure dose. The 10 µM dose, defined as a high dose, was tested as a reference to the concentration used in several in vitro studies that observed BPS effects with it for acute exposure in ovarian follicular cells [27,37]. On the other hand, all conditions in our study (including the control condition) contained the same ethanol concentration (1/10,000 = 0.01%). For each culture, Petri dishes containing droplets of 100 μL of culture medium covered with mineral oil were pre-equilibrated overnight at 38.5 °C in 5% CO_2_ in air under 95% relative humidity. Isolated measured healthy follicles were washed twice in TCM199+ and then randomly allocated into the three treatment groups. Follicles were individually placed into each 100 μL droplet and incubated for up to 15 days. At days D6 and D13, 50% of the culture medium in each droplet was replaced with fresh pre-incubated medium, and the medium removed from each droplet was individually frozen at −20 °C for the hormone assays, as described below. The 15-day culture period was optimal for studying in vitro basal follicular growth of early antral follicles and the antrum appearance in this in vitro model [58]. Indeed, in this model, the antral cavity appears in follicles with a diameter ≥320 µm, a size obtained after 6 days of culture. Furthermore, at D15 the follicles reach a size of about 550 µm and about 80% of these follicles have an antrum.

### 2.2. Morphological Evaluation of Follicles

Morphological evaluation occurred at D6, D13 and D15, while manipulating follicles on a heating plate (38 °C) for the least amount of time. Follicle morphology was assessed on seven independent experiments (each with eight follicles per condition) using three criteria: (1) follicular survival, (2) follicular growth determined by their diameter and (3) the formation of an antral cavity, defined as a visible, translucent area within the follicular cell mass. These parameters were measured only in healthy follicles: a follicle was considered healthy when it was an intact follicle (no breakdown of the basal lamina and extrusion of the oocyte) with a light oocyte and a measurable growth within one week.

### 2.3. In Vitro Follicular Hormonal Secretion Assays

For hormonal quantification, 50 µL and 70 µL of culture medium on the 100 µL of each droplet (in which individual follicle was cultured) was recovered at D13 and D15 respectively from each alive follicle, meaning it had maintained structural integrity and growth. The concentrations of AMH, oestradiol and progesterone in the culture medium were determined for 32–37 individual follicles per treatment at either D13 or D15 (as described below).

#### 2.3.1. Anti-Müllerian Hormone

The AMH concentration in the culture media at D15 was determined in 50 μL of culture medium diluted to 1/15 (in MEM+) using the AMH Gen II ELISA kit (Beckman Coulter, Villepinte, France), which had previously been validated for the analysis of ovine samples [60]. In our working conditions, the limit of detection of the assay was 78 pg/mL and the intra-assay coefficients of variation (CV) were 11.1% for an AMH concentration of 78 pg/mL and <5% for AMH concentrations >1250 pg/mL.

#### 2.3.2. Oestradiol

The oestradiol concentration in the culture media at D13 was determined using the E2-EASIA immunoassay kit (DIAsource, Louvain-la- Neuve, Belgium) from the 50-μL aliquots of culture medium, diluted to 1/5 or to 1/10, as described previously [58]. For the present assay, the limit of detection was 3 pg/mL and the intra-assay CVs were <3.5% for oestradiol concentrations ranging from 3 to 100 pg/mL.

#### 2.3.3. Progesterone

The progesterone concentration was determined in 50 µL of undiluted culture medium at D15 using a previously described ELISA protocol [61]. The limit of detection of the assay was 0.25 ng/mL for a 10 µL deposit volume and the intra-assay CVs averaged 7.1% for progesterone concentrations ranging from 0.25 to 32 ng/mL.

### 2.4. Gene Expression Analysis

#### 2.4.1. RNA Extraction and Reverse Transcription

A total of 64 alive follicles at D15 (from four independent experiments, 18–24 follicles per conditions) were used for the evaluation of gene expression and individually stored in 20 μL lysis buffer for RNA extraction at −80 °C until use. The diameter of these follicles was 300–870 μm; 46 presented an antrum and 18 did not.

Total RNA was extracted using the Nucleospin RNA XS kit (Macherey Nagel, Hoerdt, France), according to the manufacturer’s instructions, including on-column DNase treatment. Total RNA was quantified using a NanoDrop ND-1000 spectrophotometer (Nyxor Biotech, Paris, France). Reverse transcription was performed with 50 ng of total extracted follicle RNA using the Maxima First Strand cDNA Synthesis Kit (Thermo-Fisher Scientific, Illkirch-Graffenstaden, France), according to the manufacturer’s recommendations.

#### 2.4.2. Quantitative PCR Amplification

Real time polymerase chain reaction (qPCR) was performed on 2 ng of cDNA, as described previously [62]. The expression of 19 genes (Table 1) was assessed; they are involved in follicle functionality (Cytochrome P450 Family 19 Subfamily A Member 1 (*CYP19A1*), Estrogen Receptor 1 (*ESR1*), Estrogen Receptor 2 (*ESR2*), Follicle-Stimulating Hormone Receptor (*FSHR*), Hydroxy-Delta-5-Steroid Dehydrogenase (*HSD3B1*), Bone Morphogenetic Protein 15 (*BMP15*) and Aryl Hydrocarbon Receptor (*AHR*), or involved in redox status (Catalase (*CAT*), Cytochrome C Oxidase Subunit 4I1 (*COX4I1*), Cytochrome C Oxidase Subunit 5B (*COX5B*), Glutathione Peroxidase 3 (*GPX3*), Glutathione Peroxidase 8 (*GPX8*), NADH Dehydrogenase Ubiquinone 1 Beta Subcomplex Subunit 4 (*NDUFB4*), NADH Dehydrogenase Ubiquinone 1 Beta Subcomplex Subunit 5 (*NDUFB5*), NADH Ubiquinone Oxidoreductase Core Subunit V2 (*NDUFV2*), NADH Ubiquinone Oxidoreductase Complex Assembly Factor 2 (*NDUFAF2*), Succinate Dehydrogenase Complex Flavoprotein Subunit A (*SDHA*), Superoxide Dismutase 1 (*SOD1*) and Superoxide Dismutase 2 (*SOD2*)). The efficiency of the primers (Table 1) and the standard curve was determined for each gene. The expression level of each candidate gene was normalized using the geometric mean of two housekeeping genes (ribosomal protein L19 (*RPL*19) and beta-actin (*ACTB*)). The relative amounts of gene transcripts (*R*) were calculated according to the following equation:

(1) $$R=\frac{\left(E_{gene}^{-Ct\;gene}\right)}{\left(geometric\;mean\;\left(E_{RPL19}^{-Ct\;RPL19};\;E_{ACTB}^{-Ct\;ACTB}\right)\right)}$$

where *E* is the primer efficiency of each primer pair and *Ct* is the cycle threshold. 

### 2.5. Statistical Analysis

GraphPad Prism 9 (Version 9.3.1, GraphPad Software, Ritme, Paris, France) was used to carry out statistical analyses. Except for the results for the follicular survival and antrum appearance data that are presented as percentages, all results are expressed as mean +/− standard error of the mean (SEM). The data were tested for normality and the homogeneity of variances with the D’Agostino and Pearson test and the Brown–Forsythe test, respectively. Based on these tests, the effects of treatments on oestradiol, progesterone and AMH secretions were analysed with the non-parametric Kruskal–Wallis test followed by a Dunn’s multiple comparison post hoc test when a significant global difference was observed. The effects of treatment on follicular diameter growth and gene expressions were analysed with the parametric Brown–Forsythe ANOVA test, and when a significant global difference was observed, Dunnett’s T3 multiple comparison test was executed. Nonparametric Spearman correlation coefficients were used to assess the correlation between gene expressions, follicle diameter at D15, antrum presence at D15, oestradiol secretion at D13 and progesterone and AMH secretions at D15. Correlations were considered significant when |r| ≥ 0.70 and *p* < 0.0001. Lastly, the differences in the percentage of follicles that presented an antrum were compared between experimental groups at D6, D13 and D15 using a multiple logistic regression analysis. The Kaplan–Meier survival curves were drawn for the three conditions throughout the experiment and compared using the log-rank (Mantel–Cox) test. Differences were considered significant when *p* < 0.05.

## 3. Results

### 3.1. BPS Effects on Ovine Follicular Survival, Follicular Growth and Antrum Appearance

**Figure 1:**
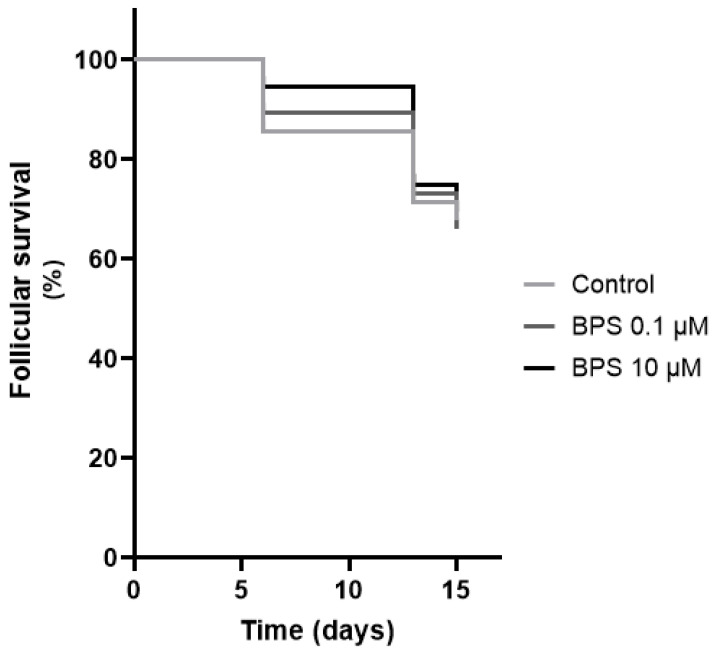
In vitro effects of bisphenol S (BPS) on ovine follicular survival. The survival of each follicle was assessed after 6, 13 and 15 days of treatment with or without BPS 0.1 µM or BPS 10 µM. A follicle was considered alive when there was no breakdown of the basal lamina and no extrusion of the oocyte. The oocyte had to be clear, and the follicular growth had to be measurable within 1 week. The results are representative of seven independent cultures with eight replicates per condition (*n* = 56 per condition). The results are expressed as percentage of alive follicles and the Kaplan–Meier survival curves obtained for the three conditions were compared using the Log-rank (Mantel–Cox) test. Differences were considered significant when *p* < 0.05.

The percentage of control alive follicles decreased from 85.7% at D6, to 71.4% at D13 and to 67.9% at D15 (Figure 1). The Kaplan–Meier survival analysis showed that the BPS 0.1 and 10 µM survival curves were not significantly different from the control one (Figure 1).

**Figure 2:**
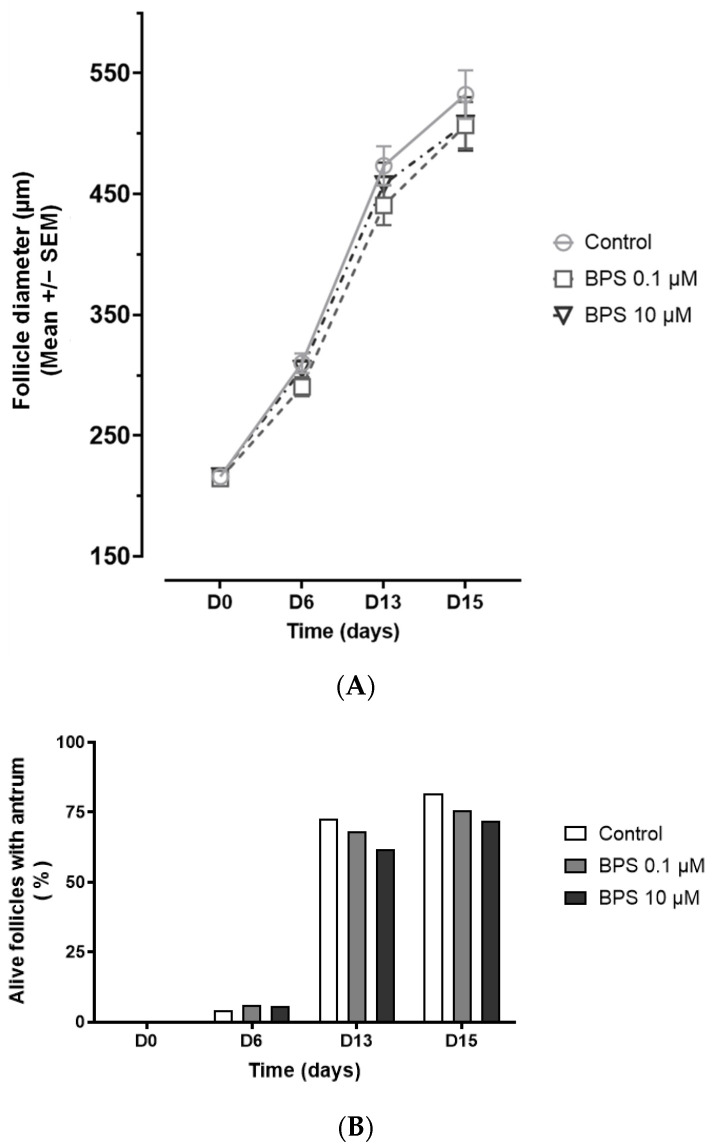
In vitro effects of bisphenol S (BPS) on ovine follicular growth and antrum appearance. The diameter evolution (**A**) and the antral cavity appearance (**B**) for each alive follicle were assessed after 6, 13 and 15 days of treatment with or without BPS 0.1 µM or BPS 10 µM. The results are representative of seven independent cultures with eight replicates per condition (*n* = 56 per condition at day 0, *n* = 48–53 according to the conditions at day 6, *n* = 40–42 according to the conditions at day 13 and *n* = 38–39 according to the conditions at day 15). For the diameter evolution, the results are expressed as mean +/− SEM and were analysed with a Brown–Forsythe ANOVA test at each day of measure (**A**). For the antrum appearance, the results are expressed as the percentage of alive follicles with an antrum and were analysed with a logistic regression at each day of measure (**B**). Differences were considered significant when *p* < 0.05.

At the beginning of the culture (D0), the diameter of the follicles (Figure 2A) was similar in the three experimental groups (216.1 +/− 2.7 µm for control, 214.6 +/− 2.6 µm for BPS 0.1 µM and 216.3 +/− 2.3 µm for BPS 10 µM, *p* = 0.88) and they presented no antral cavity (Figure 2B). After 6, 13 and 15 days of culture, the control follicles were 310 +/− 8.0 µm, 473.5 +/− 16.0 µm and 532.4 +/− 20.2 µm in diameter, respectively. There was no significant difference in the follicular diameter growth between conditions at any time point (Figure 2A).

The percentage of control alive follicles with an antral cavity increased from 4.2% at D6, to 72.5% at D13 and to 81.6% at D15 (Figure 2B). Neither BPS treatment affected the percentage of follicles presenting an antrum for each measurement day.

### 3.2. BPS Effects on Ovine Follicular Hormonal Secretions: Oestradiol, Progesterone and AMH

**Figure 3:**
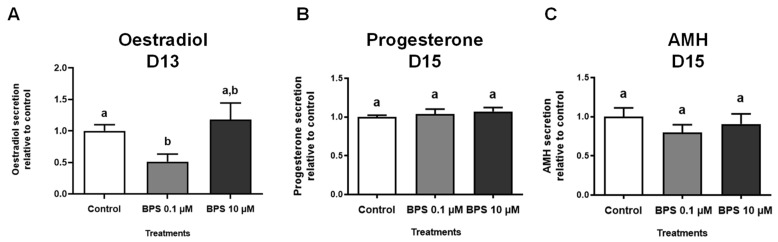
In vitro effects of bisphenol S (BPS) on ovine follicular hormonal secretions. Ovine follicles were cultured for 15 days with or without BPS (0.1 or 10 µM). Hormonal secretions were measured by ELISA in spent culture media of alive follicles after 13 days (D13) of treatment for oestradiol (**A**) and after 15 days (D15) for progesterone (**B**) and AMH (**C**). The results are representative of six independent cultures with eight replicates per condition at the beginning of the experiment for oestradiol (**A**), *n* = 34–37 alive follicles according to the conditions at day 13 and *n* = 32–33 according to conditions for progesterone (**B**) and AMH (**C**) at day 15. The data are expressed as mean +/− SEM relative to controls and were analysed with a Kruskal–Wallis test followed by a Dunn’s multiple comparison post hoc test. Bars without at least one common letter (a,b) are significantly different (*p* < 0.05).

Hormonal secretions were measured in spent culture media of alive follicles with BPS (0, 0.1 and 10 µM) for oestradiol after 13 days (Figure 3A) and for progesterone and AMH after 15 days (Figure 3B,C).

After 13 days of culture, the oestradiol concentration in the control culture medium was 108.3 +/− 11.8 pg/mL (Figure 3A). BPS 10 µM had no effect on the oestradiol concentration, but BPS 0.1 µM decreased its secretion by 48.8% compared with the control group (*p* = 0.0004, Figure 3A).

After 15 days of treatment, the progesterone and AMH concentrations in the control culture media of control group were 177.6 +/− 0.007 pg/mL (Figure 3B) and 13,943 +/− 1854 pg/mL (Figure 3C), respectively. There were no differences in these hormone levels among the three experimental groups (Figure 3B,C).

### 3.3. BPS Effects on Ovine Basal Stage Gene Expressions after 15 Days Treatment

**Figure 4:**
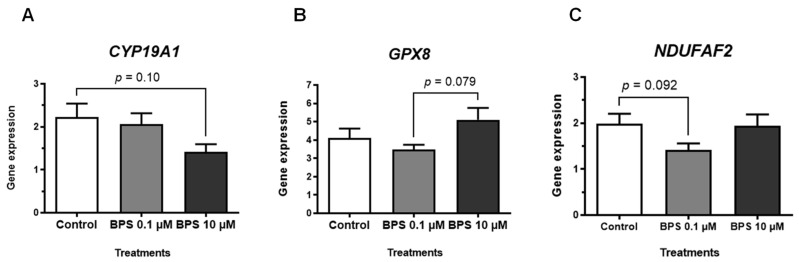
In vitro effects of bisphenol S (BPS) on ovine basal stage follicular gene expression. Ovine follicles were cultured for 15 days with or without BPS (0.1 or 10 µM). At day 15, the culture was stopped, and 64 alive follicles were used to assess the expression of 19 gene; they were preserved in a lysis buffer for RNA extraction and stored at −80 °C until use. The results are representative of four independent cultures, with *n* = 18 for control, *n* = 22 for BPS 0.1 µM and *n* = 24 for BPS 10 µM. The geometric mean of two housekeeping genes (beta-actin (*ACTB*) and ribosomal protein L19 (*RPL19*)) was used to normalise gene expression. The data are expressed as mean +/− SEM and were analysed with the Brown–Forsythe ANOVA test followed by Dunnett’s T3 multiple comparison post-hoc test. In this figure, the results are presented for the 3 genes for which a *p* value ≤ 0.10 was obtained with the Brown–Forsythe ANOVA: one gene involved in follicular development, Cytochrome P450 Family 19 Subfamily A Member 1 (*CYP19A1*, *p* = 0.064, (**A**) and two genes involved in redox status, Glutathione Peroxidase 8 (*GPX8*, *p* = 0.068, (**B**) and NADH Ubiquinone Oxidoreductase Complex Assembly Factor (*NDUFAF2*, *p* = 0.097, (**C**). The *p* values ≤ 0.10 obtained with the Dunnett’s T3 multiple comparison post-hoc test are drawn between conditions on each graph.

The expression of seven genes involved in follicular development (*CYP19A1*, *ESR1, ESR2*, *FSHR*, *HSD3B1*, *BMP15* and *AHR*) and 12 genes involved in redox status (*CAT*, *COX4I1*, *COX5B*, *GPX3*, *GPX8*, *NDUFB4*, *NDUFB5*, *NDUFV2*, *NDUFAF2, SDHA*, *SOD1* and *SOD2*) was analysed in ovine follicles after 15 days of treatment with or without BPS (0.1 or 10 µM) (Figure 4 and Table 2). No significant differences were observed for any of the analysed genes between control and BPS conditions in this study. For the total of 64 alive follicles that were analysed for gene expression, the follicular diameter at D15 was similar for all groups: 509.4 +/− 29.0 µm, 514.1 +/− 26.9 µm and 479.6 +/− 29.4 µm for the control, BPS 0.1 µM and 10 µM groups, respectively (*p* = 0.63). In addition, the percentage of follicles with an antrum at D15 was similar among the groups: 77.8% for control, 72.7% for BPS 0.1 µM (*p* = 0.71) and 66.7% for BPS 10 µM group (*p* = 0.43).

We evaluated correlations between gene expressions, D15 follicular diameter, antrum presence at D15, oestradiol secretion at D13 and progesterone and AMH secretions at D15 were studied for these 64 alive follicles (Supplementary Excel File 1, tab ‘Spearman r’ for the Spearman correlation coefficients and Supplementary Excel File 1, tab ‘*p* values’ for the associated *p* values). The results revealed several positive correlations (*p* < 0.0001) between D15 AMH secretion and D15 follicular diameter (r = 0.84), between FSHR expression and D15 follicular diameter (r = 0.70), between FSHR expression and D15 AMH secretion (r = 0.72), between AHR expression and SOD2 expression (r = 0.74) and between the expressions of several genes involved in redox status (NDUFB4–NDUFB5, r = 0.91; GPX8–NDUFB4, r = 0.82; GPX8–NDUFB5, r = 0.78; NDUFB5–SOD1, r = 0.78; NDUFB4–SOD1, r = 0.74; GPX8–SOD1, r = 0.70). There was only one negative correlation, namely between AHR expression and D15 follicular diameter (r = −0.70). There were no significant correlations between the presence of an antrum at D15 and the expression of the 19 genes.

## 4. Discussion

We aimed to evaluate the effects of BPS, currently the main substitute of BPA, on ovine follicular development and hormonal secretions during in vitro basal folliculogenesis. We have reported for the first time in a mono-ovulatory species model that a long-term exposure to a possible human exposure dose of BPS decreases oestradiol secretion by basal follicles without affecting their viability, growth and antral formation.

### 4.1. BPS Disrupted Oestradiol Secretion without Impairing Progesterone Secretion

We found that 0.1 µM BPS strongly decreased ovine oestradiol secretion (almost two-fold) after 13 days of treatment compared with the control. In sheep and humans, in vitro studies on the BPS effects on steroid secretions by ovarian somatic follicular cells has been conducted, but only on primary-cultured granulosa cells from antral follicles (2–6 mm) and preovulatory follicles, respectively [27,37]. At the concentration of 0.1 µM, the authors observed no effect of BPS on oestradiol secretion after 48-h exposure, but they found alteration of estradiol secretion from 10 µM BPS in ewes (an increase [37]) and for 50 µM BPS in humans (a decrease, [27]). In another mono-ovulatory species, the cow, oestradiol secretion from granulosa cells from antral follicles (3–7 mm) was also not affected after 6-day treatment with 0.1 µM BPS, whereas it was increased with 100 µM BPS [31]. These results, along with our data suggest that follicular cells could be more sensitive in vitro to a longer duration of BPS treatment regardless of the folliculogenesis phase, or they could be more sensitive to a possible human exposure BPS concentration (0.1 µM) for oestradiol secretion during basal folliculogenesis rather than during terminal folliculogenesis. They also show different responses of granulosa cells for oestradiol secretion according to species or the folliculogenesis phase. Furthermore, our results are consistent with a previous in vivo study from our team, which revealed a decreased oestradiol plasma level in ewes with food-restricted intakes and exposed to BPS (50 µg/kg body weight (bw)/day through diet, for at least 3 months; [63]). This decline in the oestradiol concentration was not observed in well-fed ewes [63]. In the present work, the metabolic status of the peri-pubertal ewes was not known, because they came from a slaughterhouse. Moreover, our data are also in agreement with a previous in vivo study conducted in female rodents with a long BPS exposure [43]. Indeed, Ijaz et al. [43] observed a decreased plasma oestradiol concentration in pre-pubertal rats treated with BPS (5 or 50 mg/kg bw; intraperitoneal administrations) for 28 days. However, these results are not in concordance with a previous study, which revealed an increased serum oestradiol level in mice after post-natal subcutaneous BPS treatment (50 µg/kg bw and 10 mg/kg bw) for 60 days [40]. These data indicate that the BPS administration route, dose, exposure period and treatment duration can influence the results on oestradiol level in females. Finally, our oestradiol secretion results are also consistent with those of Hu et al. [64], who examined human samples. They found a negative association between the urine BPS concentration and the serum oestradiol level in children (6–11 years) and adolescents (12–19 years) of both sexes (*n* = 1179) in the US NHANES study from 2013 to 2016. Their results were more pronounced in pubertal children. In contrast, Gao et al. [65] did not report a correlation between serum BPS and oestradiol concentrations in a population of 328 adult men and women (21–76 years) in a dense industrial area in China in 2017. These discrepancies between the studies could be linked to the age of the subjects (children versus adults) or to the level of BPS exposure, which was lower in the Chinese study (0.072 ng/mL) than in the US NHANES study (2.02 ng/mL), even if the type of biological fluid should be taken into account. An interesting point in connection with the study by Hu et al. [64] is that biological material for our experimental model of follicular development comes from peri-pubertal sheep ovaries. It could raise the question of whether there is greater BPS sensitivity of ovarian somatic follicular cells during the peri-pubertal period.

Unlike the 0.1 µM dose, 10 µM BPS (a high concentration) had no effect on oestradiol secretion after 13 days of exposure, whereas it increased 48-h oestradiol secretion from ewe granulosa cells [37] and had no effect in human granulosa cells [27], both from large antral follicles. Although these discrepancies could be partially explained by several factors (species, cellular models and exposure durations), our results highlight a non-monotonic response of BPS, as it has been described for BPA [66]. Therefore, additional experiments are required to investigate the effects of more BPS doses, including lower exposure doses on oestradiol secretion during basal folliculogenesis and whether the same observations could be made with ovaries from adult ewes.

Regarding progesterone secretion, we have shown here no effect of the two BPS concentrations during basal folliculogenesis after 15 days of exposure. In contrast, the plasma progesterone level was decreased in female rats treated with BPS during the neonatal period (50 mg/kg bw; [42]) or the adult stage (0.5, 5 or 50 mg/kg bw; [43]). In studies on cultured granulosa cells punctured from large antral follicles, there was no effect of 0.1 µM BPS in several mono-ovulatory species, namely ovine [37], bovine [31] and human [27], findings consistent with our results. However, 10 µM BPS decreased progesterone secretion in ewe [37] and human female [27] granulosa cells, whereas it had no effect on cow granulosa cells [31], as in our study. These data highlight differences related to species, study models or to exposure durations, while also suggesting that somatic follicular cells during basal folliculogenesis could be less sensitive to BPS for progesterone secretion than during terminal folliculogenesis or pregnancy. This could be explained at least in part by the fact that progesterone production is even lower during basal folliculogenesis compared with terminal folliculogenesis or pregnancy.

Our steroid hormone secretion data support that BPS at a possible human exposure dose may disrupt oestradiol secretion during basal folliculogenesis. Oestradiol is well known to be involved in feedback regulation of gonadotrophin secretions (LH and FSH). However, it also participates directly or indirectly in enhancing folliculogenesis through autocrine-paracrine modes on granulosa cells: it increases proliferation, differentiation, the expression of growth factors and receptors (Insulin-like Growth Factor-1 (IGF-1)) gonadotrophin receptors) and the number of gap-junctions, and it attenuates apoptosis [67,68]. Therefore, our results suggest that BPS could ultimately affect antral follicular development through oestradiol regulation, but a longer follow-up of our model of follicular development is required to investigate this eventuality.

### 4.2. BPS Did Not Impair Anti-Müllerian Hormone (AMH) Secretion

In the present study, at D15 control follicles reached the early antral follicle stage (diameter of 532.4 +/− 20.2 µm). Moreover, we found a strong positive correlation (r = 0.84) between AMH secretion and follicular diameter at D15, as expected for small antral follicles and in this in vitro model of basal follicular growth [58]. In humans as in sheep, AMH is expressed in granulosa cells from growing follicles after activation of the primordial follicle and transition to the primary follicle stage [69,70]. AMH expression being maximal in pre-antral and early antral follicles, it is considered as a marker of the small antral follicle population and plays a key role in controlling ovarian reserve [1,71].

We observed no significant changes in AMH secretion after 15 days of BPS exposure in our model of in vitro basal folliculogenesis. Such absence of effect of BPS on plasma AMH level after a 3-month exposure of BPS in diet (4 and 50 µg/kg bw/day) has already been reported in ewes [36]. Moreover, a 24-h BPS treatment during oocyte maturation (0.05 mg/mL [around 200 µM]) in bovine cumulus cells and cumulus oocyte complexes (COC) had no effect on AMH mRNA and protein expressions [72]. On the contrary, BPA decreased AMH mRNA level in COC and AMH protein expression in these bovine cumulus cells [72]. BPA exposure (5 and 500 µg/kg bw/day) also decreased the serum AMH concentration in sexually mature mice [73] and BPA concentration in urine or follicular fluid was negatively correlated with the serum AMH level in women [70,71]. However, an absence of correlation was also reported in infertile women [74]. These initial data on BPS suggest that BPS and BPA could act differently on AMH production and BPS would thus affect early follicle development, independently from AMH. Additional investigation is required to confirm this possibility.

### 4.3. BPS Did Not Impair Follicular Survival, Follicular Growth and Antral Formation

In our in vitro model of basal follicular development, neither follicular survival, nor follicular growth (diameter size) nor antrum appearance were significantly affected by both doses of BPS after 6, 13 and 15 days of exposure. In our in vitro model of basal follicular development up to small antral follicles (<600 µm), follicular growth is essentially due to proliferation of granulosa cells as it is the case in vivo [1].

In agreement with our results, several in vitro studies in mono-ovulatory species have shown no effect of BPS (including 0.1 or 10 µM) on the viability and/or proliferation of granulosa cells (from large antral follicles) after 48-h exposure in ewes [37] and women [27] and after 6-day treatment in cows [31]. However, regarding viability, some in vivo studies in rodents have revealed that BPS decreased the number of antral follicles (early/small and late/large) and increased the number of atretic follicles after a 28-day treatment (5 and/or 50 mg/kg body weight/day [43]) or a 10-day neonatal exposure (5 and 50 mg/kg body weight/day; [42]). We only investigated the transition between the pre-antral and early antral stages, and we did not have information about the antral-follicle stage (>550 µm). As our model enables a 21D culture of follicles that could reach up to 1 mm [58], it could be interesting to further study follicle survival in bigger antral follicles.

Finally, some studies in rodents have reported an increased number of primordial follicles after BPS exposure [35,75]. In parallel, fewer primary [41] or secondary follicles [35] have been observed. These data emphasise that it would be necessary to investigate BPS effects on earlier basal folliculogenesis (before the pre-antral stage) in mono-ovulatory species.

### 4.4. BPS Did Not Impair mRNA Expression of Key Markers of Follicular Development

As we observed a decreased oestradiol secretion after 0.1 µM BPS treatment, we analysed the mRNA expression of the steroidogenic enzymes, *HSD3B1* and *CYP19A1*. HSD3B1 protein is involved in synthesis of progesterone and androgens in converting pregnenolone into progesterone and androstenediol into testosterone, respectively. CYP19A1 protein is essential for estrogen production; it allows the conversion of testosterone into oestradiol and androstenedione into estrone. In our study, BPS at 0.1 and 10 µM did not significantly affect the mRNA expression of *HSD3B1* and *CYP19A1*. These results for *HSD3B1* expression are consistent with the absence of BPS effect on progesterone secretion in our model. One study found a decreased expression of *CYP19A1* in swine cumulus cells after a 48-h exposure with BPS at 30 µM [30], whereas other studies have shown no correlation between the oestradiol concentration in biological fluids or in cell culture medium and mRNA and/or protein expression of steroidogenic enzymes [37,40]. Thus, it could be interesting to measure the activities of steroidogenic enzymes, as a change in their activities could lead to hormonal imbalance. Moreover, because progesterone secretion was not affected by BPS treatment in our model, it might still be important to control the expression and activities of CYP17A1 and HSD17B, which are involved in androgen production independently of progesterone synthesis.

Here, we showed no modulation of mRNA expression of *BMP15* (a specific gene of oocyte activity [1,2]), *FSHR* (a marker of follicle maturation [1,2]) and *AHR* (a possible modulator of follicular growth and steroidogenesis [76]) in the presence of BPS. Consistently, BPS (10 nM, 1 µM) had no effect on *FSHR* expression in ewe cumulus cells from COC after 6-h in vitro maturation [77]. To our knowledge, no other studies are available on the effects of BPS on the *BMP15* and *AHR* expression. In view of the few studies available on all the expression of these genes, further investigation is required to conclude definitively on the effects of BPS on the expression of development markers of oocyte and somatic follicular cells in mono-ovulatory species.

Finally, the two tested BPS concentrations had no effect on *ESR1* and *ESR2* expression in our model of basal folliculogenesis. In agreement with our results, two studies using BPS concentrations close to those tested here found no modulation of *ESR1* and *ESR2* expression in ewe cumulus cells after 6 h of in vitro maturation [77] and in human granulosa cells from pre-ovulatory follicles after 48 h of exposure [27]. In contrast, *ESR1* and *ESR2* expression was increased in ovine granulosa cells from antral follicles after 48-h treatment but only for very high doses (50 and/or 100 µM; [37]). Oestrogen receptors play an important role in ovarian follicles in supporting granulosa cell differentiation, follicular growth and oocyte maturation [78,79]. Thus, our data suggest that BPS could not affect the possibility of follicular cells to respond to oestrogens during basal folliculogenesis. Nevertheless, we have to keep in mind that BPS can activate nuclear oestrogen receptors [80,81], leading to potential modulations of estrogen signalling pathways in addition to its alteration of estradiol secretion observed in this study.

### 4.5. BPS Did Not Impair mRNA Expression of Key Players of Redox Status

Several studies have focussed on oxidative stress as a potential mechanism of action of bisphenols in different cell types [54,82,83]. Thus, we measured mRNA expression of some actors in redox-status regulation, as enzymes involved in defence against free radicals (*SOD1*, *SOD2*, *CAT*, *GPX3* and *GPX8* [84]), and several enzymes of the mitochondrial respiratory chain complexes (the NADH ubiquinone oxidoreductase for complex I, namely *NDUFB4*, *NDUFB5*, *NDUFV2* and *NDUFAF2* [85]), succinate dehydrogenase for complex II (*SDHA* [86]) and cytochrome c oxidase for complex IV (*COX4I1*, *COX5B* [87]). BPS exposure at the two tested concentrations did not significantly modulate the expression of these genes in our model of basal follicular development. Few studies are available in the literature regarding the role of BPS in oxidative stress in ovarian cells. In agreement with our results, BPS (from 10^−9^ to 10^−4^ M) had no effect on 4-h reactive oxygen species (ROS) production and 24–48-h SOD1 and SOD2 mRNA expression in a human granulosa cell line (COV434; [88]). However, the other studies have shown that BPS increased ROS production and/or decreased activities of SOD and/or CAT in ovarian cells in vivo [43] or in vitro [38,55] but mainly for high doses. Thus, it could be interesting to test other BPS doses and to measure the ROS level and the activities of these enzymes of redox-status regulation in our model, to determine whether oxidative stress is really a key mechanism of action of BPS at possible human exposure doses during follicular development.

On the other hand, we did not investigate the potential effects of BPS on DNA methylation in our in vitro model of basal follicular development. However, it could be very interesting to further investigate this mechanism of action, as it was well described for BPA [49,50,51].

### 4.6. Limitations and Strengths of the Study

Some limitations occurred in this study, related to the use of this ovine model of basal follicular growth. As mentioned above, sheep ovaries came from a local abattoir and the metabolic and health status of animals was not known. However, this status may influence BPS effects on steroid secretion [63].

Nevertheless, we chose the ewe as a translational experimental model to study ovarian human basal folliculogenesis for several reasons. Unlike rodents, the appearance of primordial follicles starts at a similar time during the gestation period in both humans and ovines [56]. Furthermore, the duration of folliculogenesis, from primordial follicle to preovulatory follicular development, is similar between the two species: nearly 170 days in ewes and 200 days in women [57] versus approximatively 20 days in rodents [3,89]. More precisely, the period of antral development is about 50 days in women and 45 days in ewes [1,57,89].

On the other hand, our study model of ovarian basal folliculogenesis is somewhat far from the physiological situation in the ovary, because follicles are cultured individually without the influence of the other follicles usually present in the ovary. However, it provides a good model to study the antrum appearance [58], which is not possible to study in vivo with the same monitoring parameters as in the present study. These cultured follicles present the main features of in vivo follicles of the same size. They are able to produce steroid hormones and AMH and to express mRNA of relevant genes (*BMP15*, *GDF9*, Zona pellucida sperm-binding protein 3 [*ZP3*], *FSHR*, *ESR1* and *ESR2)* [58]. Furthermore, we observed positive correlations between D15 follicular diameter and *FSHR* expression and AMH secretion that demonstrates a consistent model of early antral follicular development for human species.

Finally, we tested in our work a long-term exposure to 0.1 µM and 10 µM concentrations of native BPS, concentrations that we have termed as a possible human exposure dose and high dose of BPS, respectively. Indeed, in human biological fluids (urine, plasma and follicular fluid), the mean BPS concentration is 10 nM, which is 10-fold lower than our lower dose. However, this dose of 0.1 µM seems to be relevant, because several studies reported BPS concentrations close to or above 100 nM for some people [19,25,59]. However, we have to keep in mind that for these people, we do not know whether their BPS exposure time is equivalent to that in our study. Furthermore, BPS is metabolised in glucuronide form in humans, which cannot be achieved in our in vitro model. Thus, human tissues could be exposed to native BPS for a shorter time than in our in vitro model. Finally, the second dose tested in our study has allowed us to compare our results with previous data—for example, studies that used the same dose for acute exposure.

## 5. Conclusions

In the present study, we showed for the first time in a mono-ovulatory species (ovine) that a long-term BPS exposure at a possible human exposure concentration drastically decreased oestradiol secretion during basal folliculogenesis in vitro. Antral formation, follicular survival and growth and AMH and progesterone secretions were not affected by BPS. The results suggest that BPS could impair oestradiol secretion early in ovarian follicular development in vivo. It may therefore affect the quality of the follicle in the long term. This work may contribute to raising the question of categorising BPS as an endocrine disruptor and of substituting BPA with BPS. It also underlines the necessity to assess the risk of BPS exposure for reproduction of mono-ovulatory mammals. Further investigation is required to confirm potential BPS effects during in vivo basal folliculogenesis and study the BPS effects on primordial follicle activation and on antral stage preceding terminal folliculogenesis and to elucidate its mechanisms of action in mono-ovulatory species.

## Figures and Tables

**Table 1:** Primer sequences for real time reverse transcription polymerase chain reaction used in this study.

Abbrev.	Gene Name	Transcript Accession Number (Ensembl)	Forward Primer (5′→3′)	Reverse Primer (5′→3′)	Size (bp)	E (%)
**Specific genes of follicle functionality**						
*AHR*	Aryl Hydrocarbon Receptor	ENSOART00020003479.1	TGGGGCTGTTTCAATGTACC	TACAGGAATCCACCGGATGT	233	81.8
*BMP15*	Bone morphogenetic protein 15	ENSOART00020018955.1	TCTATTGCCCACCTGCCTGAG	TGAAGCTGATGGCGGTAAACC	326	89.2
*CYP19A1*	Cytochrome P450 Family 19 Subfamily A Member 1	ENSOART00020040485.1	GGTCATCCTGGTCACCCTTCTG	GCCGGTCGCTGGTCTCGTCTGG	119	100
*ESR1*	Estrogen receptor 1	ENSOART00020034283.1	CCAGTTCCTCCTCCTCCTCT	GGCTCTGATTCACGTCTTCC	158	87.2
*ESR2*	Estrogen receptor 2	ENSOART00020022015.1	ACTATGGAGTCTGGTCAT	GTCGGTTCTTATCTATGGTA	114	97.3
*FSHR*	Follicle-stimulating hormone receptor	ENSOART00000004728.	GGGCCAAGTCAACTTACCACT	TGCAAATTGGATGAAGGTCA	144	88.5
*HSD3B1*	Hydroxy-Delta-5-Steroid Dehydrogenase	ENSOART00020002039.1	TCATTGACGTCAGGAATGCT	CTCTATGGTGCTGGTGTGGA	128	84
**Specific genes of redox status**						
*CAT*	Catalase	ENSOART00020018520.1	GAAACGCCTGTGTGAGAACA	AGCTTTCTCCCTTGCAGACA	208	91.2
*COX4I1*	Cytochrome C Oxidase Subunit 4I1	ENSOART00020014820.1	AGAGCTTTGCCGAGATGAAC	TCATGTCGAGCATCCTCTTG	182	88.3
*COX5B*	Cytochrome C Oxidase Subunit 5B	ENSOART00000014875.1	GGGCTAGAGAGGGAGGTCAT	CAGCCAGAACCAGATGACAG	180	91.2
*GPX3*	Glutathione Peroxidase 3	ENSOART00020022210.1	GATGTGAACGGGGAGAAAGA	CCCACCAGGAACTTCTCAAA	152	90.4
*GPX8*	Glutathione Peroxidase 8	ENSOART00020019722.1	AAGGCATTTGCAGTCTTGCT	GACCTTCAGGGTTGACCAGA	101	85.3
*NDUFB4*	NADH Dehydrogenase Ubiquinone 1 Beta Subcomplex Subunit 4	ENSOART00020003605.1	GGCCAGCCTACCTACTACCC	TGCATAGGTCCAACGAATCA	181	90.7
*NDUFB5*	NADH Dehydrogenase Ubiquinone 1 Beta Subcomplex Subunit 5	ENSOART00020014020.1	GATTGCCCGAACTTTCTTTG	AGTGCCTTATCGATGGTTGG	174	82.1
*NDUFV2*	NADH Ubiquinone Oxidoreductase Core Subunit V2	ENSOART00020029984.1	TCGAAAGCCTGTTGGAAAGT	ACACCAAACCCAGGTCCTTT	205	60.8
*NDUFAF2*	NADH Ubiquinone Oxidoreductase Complex Assembly Factor 2	ENSOART00020010561.1	AACAGAATGGGAAGCTTGGA	AGAGGCGTGCCCTTTAATCT	196	84.2
*SDHA*	Succinate Dehydrogenase Complex Flavoprotein Subunit A	ENSOART00000016992.1	AGCAGAAGAAGCCGTTTGAG	TCGGTCTCGTTCAAAGTCCT	121	93.3
*SOD1*	Superoxide Dismutase 1	ENSOART00020002019.1	CAAAAATGGTGTTGCCATTG	CCAGCGTTTCCAGTCTTTGT	153	94.0
*SOD2*	Superoxide Dismutase 2	ENSOART00020009379.1	GGTTGGCTTGGCTTCAATAA	ACATTCCAAATGGCCTTCAG	178	90.6
**Housekeeping** **genes**						
*ACTB*	Beta Actin	ENSOART00020013384.1	CCAGCACGATGAAGATCAAG	ACATCTGCTGGAAGGTGGAC	102	97.2
*RPL19*	Ribosomal Protein L19	ENSOART00020024842.1	CACAAGCTGAAGGCAGACAA	TGATGATTTCCTCCTTCTTGG	129	95.3

Abbrev.: gene name abbreviation; bp: base pair; E: efficiency.

**Table 2:** In vitro effects of bisphenol S (BPS) on ovine basal stage follicular gene expressions.

Gene Name Abbreviation	Gene Expression (Mean +/− SEM)			*p*
	Control	BPS 0.1 µM	BPS 10 µM	
**Specific genes of follicle functionality**				
*AHR*	0.986 +/− 0.282	0.808 +/− 0.194	1.260 +/− 0.232	0.367
*BMP15*	3.806 +/− 0.629	3.938 +/− 0.721	5.757 +/− 1.067	0.180
*ESR1*	0.533 +/− 0.074	0.547 +/− 0.056	0.621 +/− 0.080	0.631
*ESR2*	4.487 +/− 0.666	3.784 +/− 0.468	4.745 +/− 0.691	0.505
*FSHR*	2.461 +/− 0.400	2.552 +/− 0.473	2.245 +/− 0.466	0.876
*HSD3B1*	0.039 +/− 0.011	0.034 +/− 0.009	0.047 +/− 0.012	0.691
**Specific genes of redox status**				
*CAT*	0.672 +/− 0.064	0.775 +/− 0.066	0.609 +/− 0.056	0.152
*COX4I1*	0.619 +/− 0.042	0.614 +/− 0.056	0.602 +/− 0.049	0.970
*COX5B*	1.291 +/− 0.151	1.288 +/− 0.124	1.284 +/− 0.160	0.999
*GPX3*	0.212 +/− 0.035	0.166 +/− 0.028	0.152 +/− 0.028	0.363
*NDUFB4*	7.039 +/− 0.754	6.002 +/− 0.472	7.056 +/− 0.635	0.393
*NDUFB5*	5.380 +/− 1.034	4.464 +/− 0.710	6.608 +/− 1.392	0.353
*NDUFV2*	2.205 +/− 0.264	2.085 +/− 0.248	2.118 +/− 0.233	0.944
*SDHA*	1.038 +/− 0.066	0.991 +/− 0.088	1.139 +/− 0.116	0.502
*SOD1*	3.517 +/− 0.362	3.387 +/− 0.234	3.853 +/− 0.316	0.518
*SOD2*	0.753 +/− 0.098	0.701 +/− 0.059	0.708 +/− 0.073	0.885

Ovine follicles were cultured for 15 days with different concentrations of BPS (0, 0.1 or 10 µM). At day 15, the culture was stopped and 64 alive follicles were used to assess the expression of 19 genes; they were preserved in lysis buffer for RNA extraction and stored at −80 °C until use. The results are presented for six genes involved in follicular development (Aryl Hydrocarbon Receptor (*AHR*)), Bone morphogenetic protein 15 (*BMP15*), Estrogen receptor 1 (*ESR1*), Estrogen receptor 2 (*ESR2*), Follicle-stimulating hormone receptor (*FSHR*) and Hydroxy-Delta-5-Steroid Dehydrogenase (*HSD3B1*)) and 10 genes involved in redox status, (Catalase (*CAT*), Cytochrome C Oxidase Subunit 4I1 (*COX4I1*), Cytochrome C Oxidase Subunit 5B (*COX5B*), Glutathione Peroxidase 3 (*GPX3*), NADH Dehydrogenase Ubiquinone 1 Beta Subcomplex Subunit 4 (*NDUFB4*), NADH Dehydrogenase Ubiquinone 1 Beta Subcomplex Subunit 5 (*NDUFB5*), NADH Ubiquinone Oxidoreductase Core Subunit V2 (*NDUFV2*), Succinate Dehydrogenase Complex Flavoprotein Subunit A (*SDHA*), Superoxide Dismutase 1 (*SOD1*) and Superoxide Dismutase 2 (*SOD2*)). The results are representative of four independent cultures, with *n* = 18 for control, *n* = 22 for BPS 0.1 µM and *n* = 24 for BPS 10 µM. The geometric mean of two housekeeping genes (beta-actin (*ACTB*) and ribosomal protein L19 (*RPL19*)) was used to normalise gene expression. The data are expressed as mean +/− SEM and were analysed with the Brown–Forsythe ANOVA test followed by Dunnett’s T3 multiple comparison post hoc test. A difference was considered significant for *p* < 0.05.

## Data Availability

The data presented in this study are available only in the present article and its supplementary Table S1.

## Supplementary Materials

The following are available online at https://www.mdpi.com/article/10.3390/toxics10080437/s1, Table S1: Correlations between gene expression, follicular diameter, antrum presence and hormonal secretions in cultured ewe basal follicles. Ovine follicles were cultured in Petri dishes for 15 days with different concentrations of BPS (0, 0.1 or 10 µM). At day 15, the culture was stopped and 64 alive follicles were used to assess the expression of 19 genes: seven genes involved in follicular development (aryl hydrocarbon receptor [*AHR*], bone morphogenetic protein 15 [*BMP15*], cytochrome P450 family 19 subfamily a member 1 [*CYP19A1*], oestrogen receptor 1 [*ESR1*], (oestrogen receptor 2 [*ESR2*], follicle-stimulating hormone receptor [*FSHR*] and hydroxy-delta-5-steroid dehydrogenase [*HSD3B1*]) and 12 genes involved in redox status, (catalase [CAT], cytochrome c oxidase subunit 4I1 [COX4I1], cytochrome c oxidase subunit 5B [COX5B], glutathione peroxidase 3 [GPX3], glutathione peroxidase 8 [GPX8], NADH dehydrogenase ubiquinone 1 beta subcomplex subunit 4 [NDUFB4], NADH dehydrogenase ubiquinone 1 beta subcomplex subunit 5 [NDUFB5], NADH ubiquinone oxidoreductase core subunit V2 [NDUFV2], NADH ubiquinone oxidoreductase complex assembly factor 2 [NDUFAF2], succinate dehydrogenase complex flavoprotein subunit A [SDHA], superoxide dismutase 1 [SOD1] and superoxide dismutase 2 [SOD2]). This file includes nonparametric Spearman correlation coefficients (tab ‘Spearman r’) and the associated *p* values (tab ‘*p* values’) that were assessed between gene expression, follicular diameter at day 15 (D15), antrum presence at D15, oestradiol secretion at D13 and progesterone and anti-Müllerian hormone (AMH) secretion at D15. Correlations were considered significant when |r| ≥ 0.70 and *p* < 0.0001.
